# Automatic Framework for Extraction and Characterization of Wetting Front Propagation Using Tomographic Image Sequences of Water Infiltrated Soils

**DOI:** 10.1371/journal.pone.0115218

**Published:** 2015-01-20

**Authors:** Dionicio Vasquez, Jacob Scharcanski, Alexander Wong

**Affiliations:** 1 Graduate Program on Computer Science and Graduate Program on Electrical Engineering, Federal University of Rio Grande do Sul, P.O. Box 15064, 91501-970, Porto Alegre, RS, Brasil; 2 Dept. of Systems Design Engineering, U. of Waterloo, Waterloo, Canada; Oregon State University, UNITED STATES

## Abstract

This paper presents a new automatic framework for extracting and characterizing the dynamic shape of the 3D wetting front and its propagation, based in a sequence of tomographic images acquired as water (moisture) infiltrates in unsaturated soils. To the best of the authors’ knowledge, the shape of the 3D wetting front and its propagation and progress over time has not been previously produced as a whole by methods in existing literature. The proposed automatic framework is composed two important and integrated modules: i) extraction of the 3D wetting front, and ii) characterization and description of the 3D wetting front to obtain important information about infiltration process. The 3D wetting front surface is segmented from 3D CT imagery provided as input via a 3D stochastic region merging strategy using quadric-regressed bilateral space-scale representations. Based on the 3D segmentation results, the normal directions at local curvature maxima of the wetting front surface are computed for 3D images of soil moisture, and its propagation is analyzed at the local directions in sites of maximal water adsorption, and described using histograms of curvature changes over time in response to sample saturation. These curvature change descriptors provide indirect measurements of moisture infiltration in soils, and soil saturation. Results using a field tomograph equipment specific for soil studies are encouraging, and suggest that the proposed automatic framework can be applied to estimate the infiltration of water in soils in 3D and in time.

## Introduction

Measuring transport properties in porous media is an important issue in many areas, especially in soils science, where knowing how moisture infiltrates in the soil has fundamental importance for soil management and conservation. There are several methods available in the literature designed to estimate the static (unsaturated) porous medium structure, such pore size distributions and pore connectivity [1] [2] [3] [4] [5]. Nevertheless, these methods were not designed to handle the dynamic features of porous media as they saturate; in other words, these methods were not designed to estimate and measure how moisture infiltrates through porous media, as it goes from an unsaturated sample to moisture saturated sample.

Detecting and describing moisture infiltration in 3D is an important step to estimate the dynamic properties of heterogeneous complex stochastic materials [6], like soils. Noninvasive techniques can be very helpful in modeling and visualizing the structure and the distribution of fluids in soils. Among the several imaging techniques to approach this problem, magnetic resonance imaging has been useful in studies of fluid transport in porous media, but quantitative measurements are challenging at low saturation levels [7]. Neutron radiography is a real-time, non-invasive, imaging method that overcomes the limitations of magnetic resonance and x-rays, and this method works especially well when the substance under study contains hydrogen, or other elements with neutron attenuating properties [8]. In fact, a method for real-time analysis for wetting front detection using neutron radiography has been proposed [9]. However, this method relies on two-dimensional images, and the wetting front is then analyzed only along the vertical direction, so full 3D wetting front shape information is not available. An interesting non-invasive technique for soil analysis was proposed for a new field computerized tomography (CT) equipment [10]. As described in [11], this CT equipment allows to acquire 3D images of soils in situ, study the infiltration process, and obtain parameter estimates such as the definition of geometrical descriptors for characterizing porous adsorbents materials by detecting adsorption sites [12]. Felipussi et al. [15] [16] proposed a graph-based scheme to model [17] void connections and porosity in tomographic images of unsaturated soils acquired with the equipment in [10], but their method can not be used to estimate the dynamics of moisture infiltration in soils. As such, in general there are few noninvasive methods in literature that can detect and describe 3D moisture infiltration.

In this paper, we present a new automatic framework for extracting and characterizing the dynamic shape of the 3D wetting front and its propagation, based in the processing of a sequence of tomographic images acquired as water (moisture) infiltrates in unsaturated soils. The shape of the 3D wetting front and its progress provides very important information to studies in soil management, soil fertilization, pollution control, erosion control, precision agriculture, to name a few areas benefiting from this research. To the best of the authors’ knowledge, the dynamic shape of the 3D wetting front and its propagation as water (moisture) infiltrates in unsaturated soils is currently not provided by methods in existing literature. This is a significant extension of our previous preliminary work on detection of wetting front surfaces, as the proposed work here builds upon the wetting front detection algorithm proposed in [20], and goes beyond that work by providing a complete, automatic framework for both extraction and characterization of wetting front characteristics at different time instances, as the soil sample increases its levels of moisture saturation.

The rest of this paper is organized as follows. First, the proposed framework is described. Experimental results to evaluate the efficacy of the proposed framework using field tomograph specific for soil studies are then presented and conclusions are drawn.

## Materials and Methods

The data set utilized in the experimental work was provided by CNPDIA-Embrapa, São Carlos, Brazil. This data set was previously utilized in the doctoral thesis, [10] (which is publicly available at http://www.teses.usp.br/teses/disponiveis/18/18139/tde-13092001-091113/pt-br.php). These soil samples correspond to purple dystrophic latosoil, extracted from horizon A in the cultivated oat field managed by CNPDIA-Embrapa, in São Carlos, Brazil. These samples have been provided for research purposes only, and to the best of the author’s knowledge, these samples were collected by CNPDIA-Embrapa and did not involve endangered or protected species, nor ethical issues.

The three-dimensional processing of CT images of infiltrated soil samples is very challenging due to the need to preserve the sample topology features, as well as to factors such as the moisture infiltration in the soil sample is a stochastic process and the artifacts exhibited in the CT imaging of these materials. Differently from the methods proposed in current literature (see the ‘Introduction’ section), the proposed framework is designed to automatically provide and characterize the shape of the 3D wetting front surface and its propagation with high sensitivity, thus handling the above mentioned challenges as described below.

**Figure 1:**
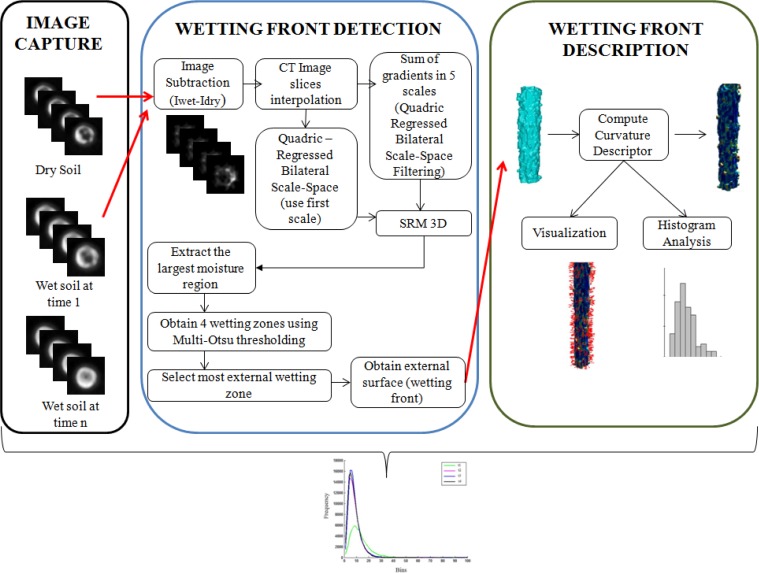
Diagram of our proposed method for 3D wetting front detection and description.

The proposed framework for 3D wetting front detection and propagation description based on CT images of moisture infiltrated soil samples is presented in the block diagram shown in Fig. 1, and all steps of the process are detailed in the ‘Three-Dimensional Wetting Front Detection’ and the ‘Description of the Detected Wetting Front’ sections.

### Three-Dimensional Wetting Front Detection

We start off by collecting dry soil sample and acquiring 3D CT images of the sample as initial input. Next, we collect four images of the soil sample in different times of the infiltration process to measure the degree of moisture infiltration. We then subtract voxel-by-voxel the sample of dry soil *I*_*dry*_ from the wet sample obtained at time *t*_*i*_ ($I_{wet}^{i}$), obtaining only the moisture contents (regions) at time *t*_*i*_, resulting in the moisture images $I_{moisture}^{i}$ at times *t*_*i*_, *i* = [1, 4]. Next, quadric-regressed bilateral scale-space decomposition [20] (see the ‘Quadric-Regressed Bilateral Scale-Space’ section) is performed on $I_{moisture}^{i}$ to obtain a scale-space representation $I_{ss}^{ij}$ corresponding to scales *σ*_*j*_, *j* = [1, 5].

**Figure 2:**
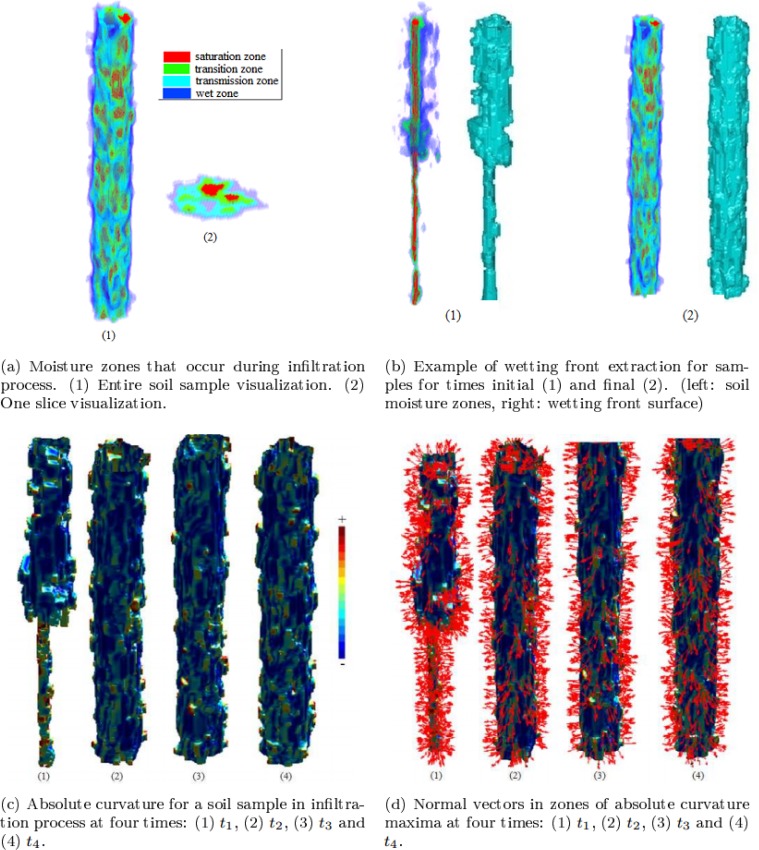
Illustration of the proposed framework for a soil sample: (a) moisture region detection; (b) wetting front extraction; (c) absolute curvature computation; and (d) normal vector computation in zones of absolute curvature maxima.

The initial CT image segmentation is based on a modified version of the Stochastic Region Merging method [14], which has been modified to work with 3D images (i.e. voxels represent gray values), with the scale-space representation as the input. In the segmentation step, $I_{ss}^{ij}$ with *i* = 1 is taken as input to calculate local differences along the *X*,*Y* and *Z* directions (see the ‘3D Stochastic Region Merging’ section). The wetting front is detected based on the 3D segmentation results ($I_{srm}^{i}$) using the Multi-Otsu thresholding method [18] and morphological operators [21]. We start by segmenting the largest moisture region from $I_{srm}^{i}$ and eliminate disconnected small regions. This largest moisture region is split into four regions using Multi-Otsu thresholding to obtain four soil moisture regions (typical of an infiltration process [19], also see the ‘Detecting Principal Moisture Zones in Soil Infiltration’ section). The wetting front is the outer boundary of the most external of the four segmented regions in $I_{m-otsu}^{i}$, here called *moisture regions*. The wetting front image $I_{WettingFront}^{i}$ is detected in the moisture region with lowest gray levels of $I_{m-otsu}^{i}$, by subtracting that moisture region from its dilation, as illustrated in Fig. 2(b) and detailed in Eqs. 1 and 2 below:

$$I_{wetzone}^{i}=\{\begin{array}{cc} 1 & \text{if}\;I_{m-otsu}^{i}(x,y,z)=min(I_{m-otsu}^{i})\\ 0 & otherwise \end{array}$$ (1)

$$I_{WettingFront}^{i}=\left\{\begin{array}{c} v(x,y,z)\in (I_{WettingSurf}^{i}-I_{m-otsu-B}^{i})\\ ,v(x,y,z)>0 \end{array}\right\}$$ (2)

where $min(I_{m-otsu}^{i})$ is the minimum gray tone in the wetting zones; $I_{wetzone}^{i}$ is a binary image with two regions, the wet zone and background; $I_{WettingSurf}^{i}$ is the binary image obtained using a subtraction and dilation operator with a structuring element of 3×3×3 voxels ($(I_{wetzone}^{i}⊗SE)-I_{wetzone}^{i}$); $I_{m-otsu-B}^{i}$ is a binary image 1,0 where the moisture regions with gray value >0 are assigned to 1; $I_{WettingFront}^{i}$ is a binary image with the wetting front assigned to 1. Finally, the volumetric reconstruction of the wetting front ($I_{WettingFront}^{i}$) is illustrated in Fig. 2(b) ((1)right and (2)right).

#### Quadric-Regressed Bilateral Scale-Space

To minimize the effects of noise in CT soil images and account for the multi-scale nature of porous media to improve accuracy, a Quadric-Regressed Bilateral Scale-Space strategy is introduced to decompose the soil images. Given $I_{moisture}^{i}$, the bilateral scale-space representation $I_{ss}^{ij}$ can be defined as in Eqs. 3, 4 and 5 [13] below ($\underline{x}=(x,y,z)$):

$$I_{ss1}^{ij}(\underline{x})=\frac{\sum_{W}\omega_{p}(\underline{x},W)\omega_{s}(\underline{x},W)I_{ss1}^{ij-1}(\underline{x})}{\sum_{W}\omega_{p}(\underline{x},W)\omega_{s}(\underline{x},W)}$$ (3)

$$\omega_{p}(\underline{x},W)=exp\left[-\frac{1}{2}{\left(\frac{\left\|I_{ss1}^{ij-1}(\underline{x})-I_{ss1}^{ij-1}(W)\right\|}{\sigma_{p}}\right)}^{2}\right]$$ (4)

$$\omega_{s}(\underline{x},W)=exp\left[-\frac{1}{2}{\left(\frac{\left\|\underline{x}-W\right\|}{\sigma_{s}}\right)}^{2}\right]$$ (5)

After scale-space decomposition, each pixel $I_{ss1}^{ij}$ is re-estimated via model-based regression within a local window *W* based on a local quadric model $I_{ss}^{ij}=p_{1}x^{2}+p_{2}y^{2}+p_{3}xy+p_{4}x+p_{5}y+p_{6}$, where the parameters *p*_*k*_ = 1, …, 6 are obtained by a least-squares fit to the data $I_{ss1}^{ij}$ within the window.

#### 3D Stochastic Region Merging (SRM3D)

The approach used for segmenting the moisture regions in the CT images is a 3D extension of the Stochastic Region Merging (SRM) [22] [23] proposed in [14]. Regions are sets of voxels with homogeneous properties, and grow iteratively by merging smaller regions with a stochastic test to decide whether regions should be merged. We assume voxels with 6-connectivity, and define a graph where each voxel *v*(*x*,*y*,*z*) initially is a node and its six neighbors are linked to *v*(*x*,*y*,*z*) by the graph edges. The edge weights are the voxel intensity differences. Initially, each voxel is assumed to be a region, and we choose to merge regions based on the predicate *α* shown in Eq. 6 [14]:

$$\alpha (R_{a},R_{b})=exp\left[-\frac{{\left(E[R_{a}]-E[R_{b}]\right)}^{2}}{\Lambda (R_{a},R_{b})}\right]$$ (6)

where *E*[⋅] is the expected value of elements (voxels) in a region (i.e. *a* or *b*), and Φ(⋅) is a statistical region merging penalty defined in Eq. 7:

$$\Lambda (R_{a},R_{b})=\frac{D_{I_{ss}}^{2}}{2Q}\left[\frac{ln(\Phi {\left(I_{ss}\right)}^{2})}{\Phi (R_{a})}+\frac{ln(\Phi {\left(I_{ss}\right)}^{2})}{\Phi (R_{b})}\right]$$ (7)

where Λ(⋅) represents the number of voxels in the argument (i.e. the representation *I*_*ss*_ or the region *R*_*a*_ or *R*_*b*_), *D*_*I*_*s*_*s*_ is the dynamic range of *I*_*ss*_ and *Q* is regularization term.

This method uses a multi-pass strategy that iteratively refines the segmentation result, assigning each voxel to a unique region. These segmented regions are subsequently merged with other regions in a stochastic manner, to yield the final segmentation result. The local difference between adjacent regions (i.e. graph edge weights) is computed using the local Difference of Gaussians (DoG) with scale *σ* = 2. In our experiments we used the regularization parameter *Q* = 100, dynamic range *D* = 256 and the number of iterations was 3.

#### Detecting Principal Moisture Zones in Soil Infiltration

According to [19], the moisture image can be partitioned in four regions: i) saturation, ii) transition, iii) transmission, and iv) wet zone. We focus on the wet zone, where there is a fast variation of moisture. The wetting front is the small external region adjacent to the wet zone where there is an abrupt variation of moisture (representing outer boundary of moisture infiltration) (Fig. 2(a)).

### Description of the Detected Wetting Front

The curvature descriptor is a powerful tool for extracting information from 3D data, [24]. Here, we show that the absolute curvature is a suitable descriptor for measuring and describing the wetting front propagation, based on reconstructed wetting front surface obtained earlier (see the ‘Three-Dimensional Wetting Front Detection’ section). The absolute curvature is carried out over the reconstructed triangular mesh from a set of tomographic images, followed by the estimation of the local curvature tensor at the vertexes of this mesh using the method proposed in [25], where after calculating the principal curvatures *K*_*min*_ and *K*_*max*_ the absolute curvature is given for *K*_*abs*_ = *abs*(*K*_*min*_)+*abs*(*K*_*max*_). This descriptor is used to identify key sites with absolute curvature maxima and to compute the normal directions, which provide information of the wetting front propagation in a given instance of time. The locations where we find these curvature maxima of the wetting front surface indicate the most favorable absorption sites of the water infiltrated soil sample (i.e., the locations where the water infiltrates more easily in the soil sample). Finally, a histogram based on curvature values for each sample in given time instance is computed to analyze the saturation state of the sample soil.

## Experiments and Results

To illustrate the performance of our proposed framework, four sequences of water infiltration in dry (unsaturated) soils were processed. Each sequence has five CT images to capture the dynamics of the water infiltration process (with the first CT image set corresponding to dry/unsaturated soil). These soil samples are of purple dystrophic latosoil, submitted to conventional treatment before planting. All are obtained at horizon A, from the first layer, near the surface at a depth in the range of 0–20 cm. The 3D images were obtained with a third-generation CT system built in the laboratories of CNPDIA-Embrapa São Carlos, Brazil. This CT system consists of a mobile personal computer PC/104, a positioning mechanism with 51 cm of diameter, a detector that consists of two PDA (photodiode array) model S6493-128G operating in the energy range of 10 to 100keV, an electronic control system for scanning, acquisition and processing of data, and an automotive battery 12V, 36 A. Each soil sample was imaged using this dedicated CT system and generated 31 slices with an inter-slice distance of 5mm, and 71 × 71 pixels of slice resolution.

**Figure 3:**
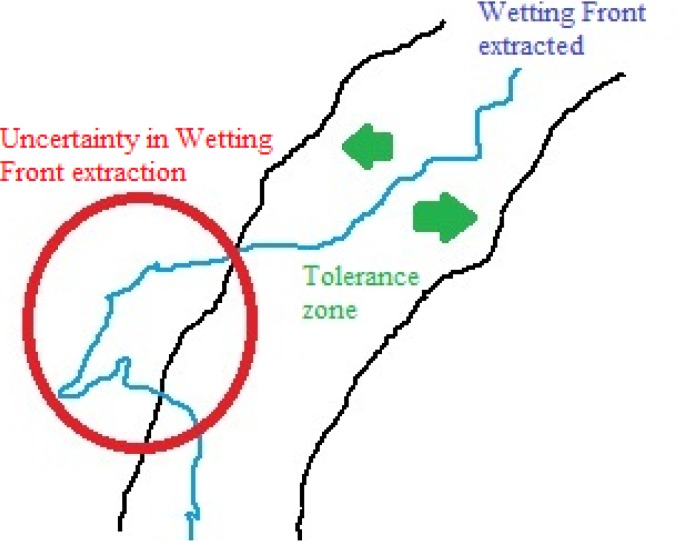
Illustration of the tolerance zone used by the proposed method to estimate the uncertainty in wetting front extraction.

In order to compare the 3D representation of the wetting front obtained for each sample of soil, a ground truth was estimated for the water infiltration CT image sequences under the supervision of an experienced person in soil image analysis at the Federal University of Rio Grande do Sul (Porto Alegre, Brazil), since it is difficult to obtain public databases and benchmarks for this type of data. We chose to estimate the set of ground truth images since it would be impractical to mark manually at the pixel level each of the 375 CT image slices, considering that each image slice has a resolution of 71 × 71 pixels. So, the tolerance zone was determined as the region with high probability of containing the wetting front, and the wetting front was determined as the outer boundary adjacent to the zone with the minimum gray levels. To refine this wetting front detection, we performed three dilations (using a structuring element of ones and size 3×3×3) on the binary image obtained with the minimum moisture content (i.e. smaller gray levels of the histogram) of the largest segmented region, as shown below:

$$I_{moisture^{B}}^{i}=\left\{\begin{array}{c} 1,H_{min}(I_{moisture}^{i}(large))\leq I_{moisture}^{i}(large)<H_{min}(I_{moisture}^{i}(large))+10;\\ 0,otherwise \end{array}\right\}$$ (8)

where *H*_*min*_(.) denotes the minimum moisture level (i.e. gray levels) in the histogram of the largest segmented region gray levels $I_{moisture}^{i}(large)$. In this way we detect the wetting front location as described in [20], and obtain as a connected region the so called tolerance zone (see Fig. 3). Based on the detected tolerance zones, the uncertainty estimation tends to be small for our framework, resulting in an average of *2.55%*, as described in Table 1. This uncertainty was calculated as the ratio between the voxels outside the tolerance zone and the total number of voxels belonging to the wetting front.

**Table 1:** Uncertainty estimation in average of the proposed method.

**Sample Id**	**Time**				**For each sample (%)**
	**t*_1_*	**t*_2_*	**t*_3_*	**t*_4_*	
PC1	1.1658	1.5108	1.6805	2.7226	1.7699
PC2	0.6667	0.3498	0.3774	2.0795	0.8683
PC3	4.6086	3.9056	3.9366	6.4201	4.7176
PC4	0.4275	2.82	6.3307	8.1547	4.4332
PC5	1.2089	0.5408	1.1717	0.9249	0.9616
**For all samples (%)**					**2.5502**

**Figure 4:**
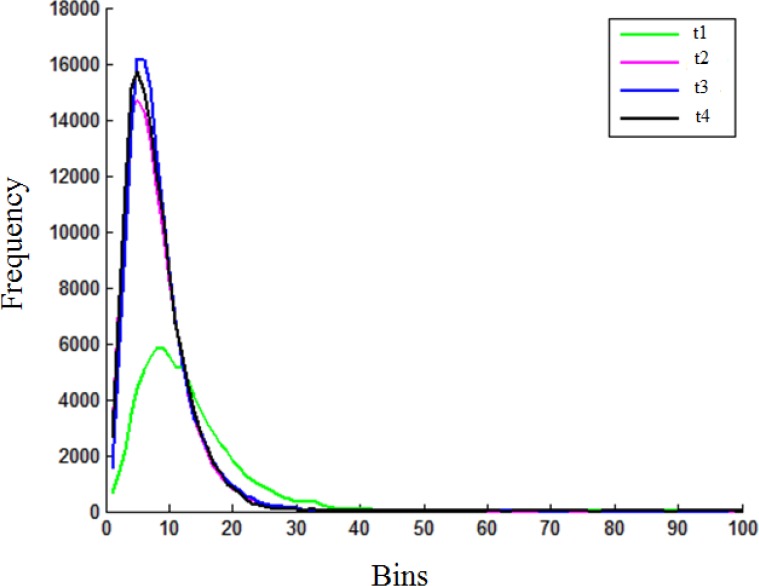
Histograms of curvature values for a sequence in infiltration process.

Since we start with a CT image set of a dry soil sample, we estimate at each time instant the 3D moisture infiltration in the soil sample as it saturates based on the differences voxel-by-voxel, since these gray level differences are due to the moisture infiltration in the soil sample. Figs. 2(c)(1–4) show the absolute curvature at different absorption sites (red indicating maximum curvature). Figs. 2(d)(1–4) show the normals to the local absolute curvature maxima, which indicate the wetting front surface propagation directions, and are calculated over the reconstructed mesh of extracted wetting front surface for each soil sample for *t*_1_ to *t*_4_ (left to right). Fig. 4 shows the histograms of curvatures for *t*_1_ to *t*_4_, and indicate the variability of absorption sites over time and are related to the sample saturation process.

In order to confirm that moisture content (volume) is in fact delimited by the detected wetting front volume, an additional experiment was performed to estimate the saturated hydraulic conductivity (*K*_*s*_) in each sample. The saturated hydraulic conductivity describes the water movement through saturated media, and is here estimated based on the geometric properties of the volume defined by the detected 3D wetting front using Eq. 9.

In fact, *K*_*s*_ has been defined in, [26] for cylindrical flow geometry. The hydraulic conductivity *K*_*s*_ has a characteristic value for each soil type, and describes how easily water (moisture) moves through the pore space. Therefore, we expect a constant *K*_*s*_ for each soil type, since it only depends on the texture and structure of the soil. Eq. 9, [26] can be used to obtain a relationship between the wetted (moisture) width (*d*), emitter discharge (*q*), the wetted (moisture) depth (*z*), and the saturated hydraulic conductivity (*K*_*s*_):

$$d=1.32z^{0.35}q^{0.33}K_{s}^{-0.33}$$ (9)

**Figure 5:**
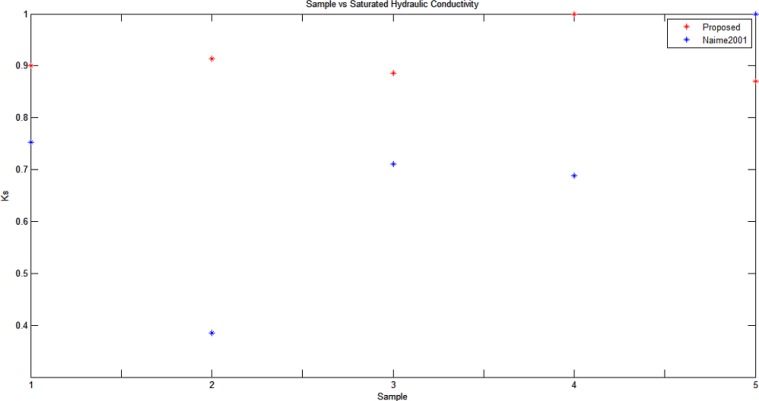
Saturated hydraulic conductivity values for five soil samples of the same soil type.

Consequently, we can define *K*_*s*_ in terms of *d*, *z* and *q*, and then verify if the obtained *K*_*s*_ values in saturated samples in fact is constant for the moisture volume delimited by the detected 3D wetting front. Since Eq. 9 is defined for a cylindrical geometry, we initially transform the saturated volume delimited by the detected 3D wetting front into a cylinder with equivalent volume, keeping the cylinder diameter (*d*) fixed, so that the equivalent volume cylinder is obtained by varying its depth (*z*) and assuming a constant arbitrary value for emitter discharge (*q*) (which approximates qualitatively the tests performed in situ). In this way, we obtain a dimensionless *K*_*s*_ value, as shown in Fig. 5. It is interesting to compare the *K*_*s*_ values estimated using the proposed framework with the *K*_*s*_ values obtained in situ in, [10] using the same soil type. It can be observed that the proposed framework tends to estimate a more robust set of *K*_*s*_ values (normalized to [0, 1]) using the above mentioned scheme, obtaining a standard deviation of 0.05 versus the standard deviation of 0.22 obtained in, [10]. Therefore, from the hydrological point of view, the moisture volume delimited by the detected 3D wetting front appears to be correct, allowing to make *K*_*s*_ estimates that are relatively constant for different samples of the infiltrated soil type, as expected considering that *K*_*s*_ is a characteristic hydrological property of the soil type.

## Conclusions

This paper presented a new integrated framework for automatically detecting the 3D shape of the wetting front surface, as well as describing and characterizing its evolution as moisture infiltrates in soil samples, using CT image sequences showing the moisture infiltration in porous media samples (soils). The dry (unsaturated) soil samples are infiltrated with a known volume of water and known rate, and the 3D wetting front surface is detected as the moisture infiltrates through the sample porous medium, and its propagation is described based on surface normals at the local curvature maxima.

Experimental results using field tomograph specific for soil studies show that the framework can be applied effectively to estimate the infiltration of water in soils in 3D and in time. Future work will focus on further evaluating the proposed wetting front propagation description, as well as its potential to provide new measurements for characterizing porous media.

## Supporting Information

S1 File The data files used on all experiments are available as supporting information (see supporting information file ‘S1’). (ZIP)
