# Supplementary figures and images for: Automatic Framework for Extraction and Characterization of Wetting Front Propagation Using Tomographic Image Sequences of Water Infiltrated Soils (part 2 of 2)
Source: PLoS One. 2015 Jan 20;10(1):e0115218. doi: 10.1371/journal.pone.0115218 (PMC4300084; doi:10.1371/journal.pone.0115218)

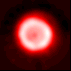

Supplement: S1 File — (ZIP) [file pone.0115218.s001.zip › TomoSolos/PC1/PC1_I_Infiltra_78.bmp]

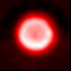

Supplement: S1 File — (ZIP) [file pone.0115218.s001.zip › TomoSolos/PC1/PC1_I_Infiltra_79.bmp]

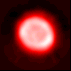

Supplement: S1 File — (ZIP) [file pone.0115218.s001.zip › TomoSolos/PC1/PC1_I_Infiltra_8.bmp]

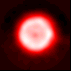

Supplement: S1 File — (ZIP) [file pone.0115218.s001.zip › TomoSolos/PC1/PC1_I_Infiltra_80.bmp]

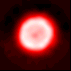

Supplement: S1 File — (ZIP) [file pone.0115218.s001.zip › TomoSolos/PC1/PC1_I_Infiltra_81.bmp]

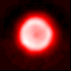

Supplement: S1 File — (ZIP) [file pone.0115218.s001.zip › TomoSolos/PC1/PC1_I_Infiltra_82.bmp]

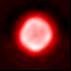

Supplement: S1 File — (ZIP) [file pone.0115218.s001.zip › TomoSolos/PC1/PC1_I_Infiltra_83.bmp]

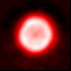

Supplement: S1 File — (ZIP) [file pone.0115218.s001.zip › TomoSolos/PC1/PC1_I_Infiltra_84.bmp]

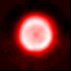

Supplement: S1 File — (ZIP) [file pone.0115218.s001.zip › TomoSolos/PC1/PC1_I_Infiltra_85.bmp]

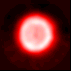

Supplement: S1 File — (ZIP) [file pone.0115218.s001.zip › TomoSolos/PC1/PC1_I_Infiltra_86.bmp]

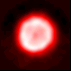

Supplement: S1 File — (ZIP) [file pone.0115218.s001.zip › TomoSolos/PC1/PC1_I_Infiltra_87.bmp]

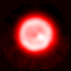

Supplement: S1 File — (ZIP) [file pone.0115218.s001.zip › TomoSolos/PC1/PC1_I_Infiltra_88.bmp]

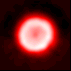

Supplement: S1 File — (ZIP) [file pone.0115218.s001.zip › TomoSolos/PC1/PC1_I_Infiltra_89.bmp]

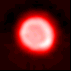

Supplement: S1 File — (ZIP) [file pone.0115218.s001.zip › TomoSolos/PC1/PC1_I_Infiltra_9.bmp]

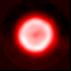

Supplement: S1 File — (ZIP) [file pone.0115218.s001.zip › TomoSolos/PC1/PC1_I_Infiltra_90.bmp]

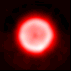

Supplement: S1 File — (ZIP) [file pone.0115218.s001.zip › TomoSolos/PC1/PC1_I_Infiltra_91.bmp]

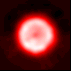

Supplement: S1 File — (ZIP) [file pone.0115218.s001.zip › TomoSolos/PC1/PC1_I_Infiltra_92.bmp]

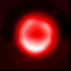

Supplement: S1 File — (ZIP) [file pone.0115218.s001.zip › TomoSolos/PC1/PC1_I_Infiltra_93.bmp]

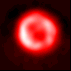

Supplement: S1 File — (ZIP) [file pone.0115218.s001.zip › TomoSolos/PC1/PC1_I_Infiltra_94.bmp]

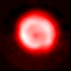

Supplement: S1 File — (ZIP) [file pone.0115218.s001.zip › TomoSolos/PC1/PC1_I_Infiltra_95.bmp]

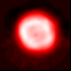

Supplement: S1 File — (ZIP) [file pone.0115218.s001.zip › TomoSolos/PC1/PC1_I_Infiltra_96.bmp]

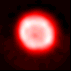

Supplement: S1 File — (ZIP) [file pone.0115218.s001.zip › TomoSolos/PC1/PC1_I_Infiltra_97.bmp]

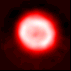

Supplement: S1 File — (ZIP) [file pone.0115218.s001.zip › TomoSolos/PC1/PC1_I_Infiltra_98.bmp]

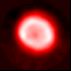

Supplement: S1 File — (ZIP) [file pone.0115218.s001.zip › TomoSolos/PC1/PC1_I_Infiltra_99.bmp]

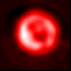

Supplement: S1 File — (ZIP) [file pone.0115218.s001.zip › TomoSolos/PC1/PC1_I_Seco1.bmp]

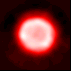

Supplement: S1 File — (ZIP) [file pone.0115218.s001.zip › TomoSolos/PC1/PC1_I_Seco10.bmp]

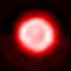

Supplement: S1 File — (ZIP) [file pone.0115218.s001.zip › TomoSolos/PC1/PC1_I_Seco11.bmp]

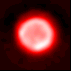

Supplement: S1 File — (ZIP) [file pone.0115218.s001.zip › TomoSolos/PC1/PC1_I_Seco12.bmp]

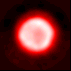

Supplement: S1 File — (ZIP) [file pone.0115218.s001.zip › TomoSolos/PC1/PC1_I_Seco13.bmp]

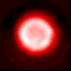

Supplement: S1 File — (ZIP) [file pone.0115218.s001.zip › TomoSolos/PC1/PC1_I_Seco14.bmp]

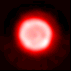

Supplement: S1 File — (ZIP) [file pone.0115218.s001.zip › TomoSolos/PC1/PC1_I_Seco15.bmp]

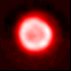

Supplement: S1 File — (ZIP) [file pone.0115218.s001.zip › TomoSolos/PC1/PC1_I_Seco16.bmp]

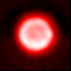

Supplement: S1 File — (ZIP) [file pone.0115218.s001.zip › TomoSolos/PC1/PC1_I_Seco17.bmp]

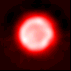

Supplement: S1 File — (ZIP) [file pone.0115218.s001.zip › TomoSolos/PC1/PC1_I_Seco18.bmp]

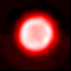

Supplement: S1 File — (ZIP) [file pone.0115218.s001.zip › TomoSolos/PC1/PC1_I_Seco19.bmp]

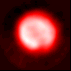

Supplement: S1 File — (ZIP) [file pone.0115218.s001.zip › TomoSolos/PC1/PC1_I_Seco2.bmp]

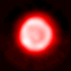

Supplement: S1 File — (ZIP) [file pone.0115218.s001.zip › TomoSolos/PC1/PC1_I_Seco20.bmp]

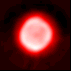

Supplement: S1 File — (ZIP) [file pone.0115218.s001.zip › TomoSolos/PC1/PC1_I_Seco21.bmp]

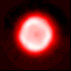

Supplement: S1 File — (ZIP) [file pone.0115218.s001.zip › TomoSolos/PC1/PC1_I_Seco22.bmp]

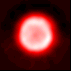

Supplement: S1 File — (ZIP) [file pone.0115218.s001.zip › TomoSolos/PC1/PC1_I_Seco23.bmp]

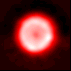

Supplement: S1 File — (ZIP) [file pone.0115218.s001.zip › TomoSolos/PC1/PC1_I_Seco24.bmp]

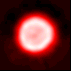

Supplement: S1 File — (ZIP) [file pone.0115218.s001.zip › TomoSolos/PC1/PC1_I_Seco25.bmp]

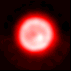

Supplement: S1 File — (ZIP) [file pone.0115218.s001.zip › TomoSolos/PC1/PC1_I_Seco26.bmp]

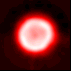

Supplement: S1 File — (ZIP) [file pone.0115218.s001.zip › TomoSolos/PC1/PC1_I_Seco27.bmp]

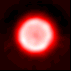

Supplement: S1 File — (ZIP) [file pone.0115218.s001.zip › TomoSolos/PC1/PC1_I_Seco28.bmp]

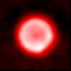

Supplement: S1 File — (ZIP) [file pone.0115218.s001.zip › TomoSolos/PC1/PC1_I_Seco29.bmp]

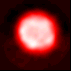

Supplement: S1 File — (ZIP) [file pone.0115218.s001.zip › TomoSolos/PC1/PC1_I_Seco3.bmp]

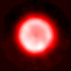

Supplement: S1 File — (ZIP) [file pone.0115218.s001.zip › TomoSolos/PC1/PC1_I_Seco30.bmp]

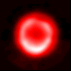

Supplement: S1 File — (ZIP) [file pone.0115218.s001.zip › TomoSolos/PC1/PC1_I_Seco31.bmp]

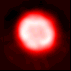

Supplement: S1 File — (ZIP) [file pone.0115218.s001.zip › TomoSolos/PC1/PC1_I_Seco4.bmp]

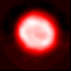

Supplement: S1 File — (ZIP) [file pone.0115218.s001.zip › TomoSolos/PC1/PC1_I_Seco5.bmp]

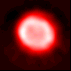

Supplement: S1 File — (ZIP) [file pone.0115218.s001.zip › TomoSolos/PC1/PC1_I_Seco6.bmp]

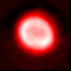

Supplement: S1 File — (ZIP) [file pone.0115218.s001.zip › TomoSolos/PC1/PC1_I_Seco7.bmp]

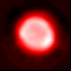

Supplement: S1 File — (ZIP) [file pone.0115218.s001.zip › TomoSolos/PC1/PC1_I_Seco8.bmp]

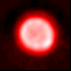

Supplement: S1 File — (ZIP) [file pone.0115218.s001.zip › TomoSolos/PC1/PC1_I_Seco9.bmp]

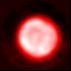

Supplement: S1 File — (ZIP) [file pone.0115218.s001.zip › TomoSolos/PC2/PC2_Infiltra_1.bmp]

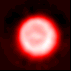

Supplement: S1 File — (ZIP) [file pone.0115218.s001.zip › TomoSolos/PC2/PC2_Infiltra_10.bmp]

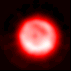

Supplement: S1 File — (ZIP) [file pone.0115218.s001.zip › TomoSolos/PC2/PC2_Infiltra_100.bmp]

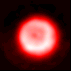

Supplement: S1 File — (ZIP) [file pone.0115218.s001.zip › TomoSolos/PC2/PC2_Infiltra_101.bmp]

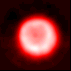

Supplement: S1 File — (ZIP) [file pone.0115218.s001.zip › TomoSolos/PC2/PC2_Infiltra_102.bmp]

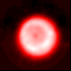

Supplement: S1 File — (ZIP) [file pone.0115218.s001.zip › TomoSolos/PC2/PC2_Infiltra_103.bmp]

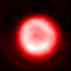

Supplement: S1 File — (ZIP) [file pone.0115218.s001.zip › TomoSolos/PC2/PC2_Infiltra_104.bmp]

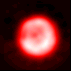

Supplement: S1 File — (ZIP) [file pone.0115218.s001.zip › TomoSolos/PC2/PC2_Infiltra_105.bmp]

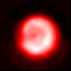

Supplement: S1 File — (ZIP) [file pone.0115218.s001.zip › TomoSolos/PC2/PC2_Infiltra_106.bmp]

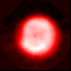

Supplement: S1 File — (ZIP) [file pone.0115218.s001.zip › TomoSolos/PC2/PC2_Infiltra_107.bmp]

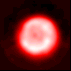

Supplement: S1 File — (ZIP) [file pone.0115218.s001.zip › TomoSolos/PC2/PC2_Infiltra_108.bmp]

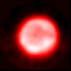

Supplement: S1 File — (ZIP) [file pone.0115218.s001.zip › TomoSolos/PC2/PC2_Infiltra_109.bmp]

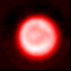

Supplement: S1 File — (ZIP) [file pone.0115218.s001.zip › TomoSolos/PC2/PC2_Infiltra_11.bmp]

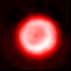

Supplement: S1 File — (ZIP) [file pone.0115218.s001.zip › TomoSolos/PC2/PC2_Infiltra_110.bmp]

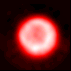

Supplement: S1 File — (ZIP) [file pone.0115218.s001.zip › TomoSolos/PC2/PC2_Infiltra_111.bmp]

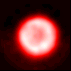

Supplement: S1 File — (ZIP) [file pone.0115218.s001.zip › TomoSolos/PC2/PC2_Infiltra_112.bmp]

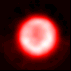

Supplement: S1 File — (ZIP) [file pone.0115218.s001.zip › TomoSolos/PC2/PC2_Infiltra_113.bmp]

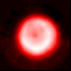

Supplement: S1 File — (ZIP) [file pone.0115218.s001.zip › TomoSolos/PC2/PC2_Infiltra_114.bmp]

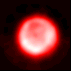

Supplement: S1 File — (ZIP) [file pone.0115218.s001.zip › TomoSolos/PC2/PC2_Infiltra_115.bmp]

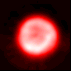

Supplement: S1 File — (ZIP) [file pone.0115218.s001.zip › TomoSolos/PC2/PC2_Infiltra_116.bmp]

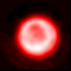

Supplement: S1 File — (ZIP) [file pone.0115218.s001.zip › TomoSolos/PC2/PC2_Infiltra_117.bmp]

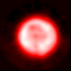

Supplement: S1 File — (ZIP) [file pone.0115218.s001.zip › TomoSolos/PC2/PC2_Infiltra_118.bmp]

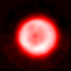

Supplement: S1 File — (ZIP) [file pone.0115218.s001.zip › TomoSolos/PC2/PC2_Infiltra_119.bmp]

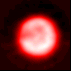

Supplement: S1 File — (ZIP) [file pone.0115218.s001.zip › TomoSolos/PC2/PC2_Infiltra_12.bmp]

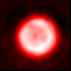

Supplement: S1 File — (ZIP) [file pone.0115218.s001.zip › TomoSolos/PC2/PC2_Infiltra_120.bmp]

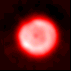

Supplement: S1 File — (ZIP) [file pone.0115218.s001.zip › TomoSolos/PC2/PC2_Infiltra_121.bmp]

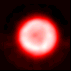

Supplement: S1 File — (ZIP) [file pone.0115218.s001.zip › TomoSolos/PC2/PC2_Infiltra_122.bmp]

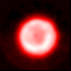

Supplement: S1 File — (ZIP) [file pone.0115218.s001.zip › TomoSolos/PC2/PC2_Infiltra_123.bmp]

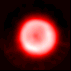

Supplement: S1 File — (ZIP) [file pone.0115218.s001.zip › TomoSolos/PC2/PC2_Infiltra_124.bmp]

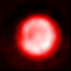

Supplement: S1 File — (ZIP) [file pone.0115218.s001.zip › TomoSolos/PC2/PC2_Infiltra_13.bmp]

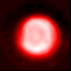

Supplement: S1 File — (ZIP) [file pone.0115218.s001.zip › TomoSolos/PC2/PC2_Infiltra_14.bmp]

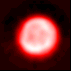

Supplement: S1 File — (ZIP) [file pone.0115218.s001.zip › TomoSolos/PC2/PC2_Infiltra_15.bmp]

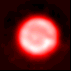

Supplement: S1 File — (ZIP) [file pone.0115218.s001.zip › TomoSolos/PC2/PC2_Infiltra_16.bmp]

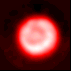

Supplement: S1 File — (ZIP) [file pone.0115218.s001.zip › TomoSolos/PC2/PC2_Infiltra_17.bmp]

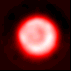

Supplement: S1 File — (ZIP) [file pone.0115218.s001.zip › TomoSolos/PC2/PC2_Infiltra_18.bmp]

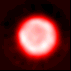

Supplement: S1 File — (ZIP) [file pone.0115218.s001.zip › TomoSolos/PC2/PC2_Infiltra_19.bmp]

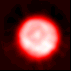

Supplement: S1 File — (ZIP) [file pone.0115218.s001.zip › TomoSolos/PC2/PC2_Infiltra_2.bmp]

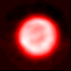

Supplement: S1 File — (ZIP) [file pone.0115218.s001.zip › TomoSolos/PC2/PC2_Infiltra_20.bmp]

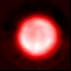

Supplement: S1 File — (ZIP) [file pone.0115218.s001.zip › TomoSolos/PC2/PC2_Infiltra_21.bmp]

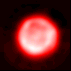

Supplement: S1 File — (ZIP) [file pone.0115218.s001.zip › TomoSolos/PC2/PC2_Infiltra_22.bmp]

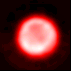

Supplement: S1 File — (ZIP) [file pone.0115218.s001.zip › TomoSolos/PC2/PC2_Infiltra_23.bmp]

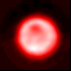

Supplement: S1 File — (ZIP) [file pone.0115218.s001.zip › TomoSolos/PC2/PC2_Infiltra_24.bmp]

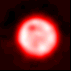

Supplement: S1 File — (ZIP) [file pone.0115218.s001.zip › TomoSolos/PC2/PC2_Infiltra_25.bmp]

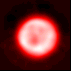

Supplement: S1 File — (ZIP) [file pone.0115218.s001.zip › TomoSolos/PC2/PC2_Infiltra_26.bmp]

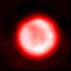

Supplement: S1 File — (ZIP) [file pone.0115218.s001.zip › TomoSolos/PC2/PC2_Infiltra_27.bmp]
