# Supplementary figures and images for: Automatic Framework for Extraction and Characterization of Wetting Front Propagation Using Tomographic Image Sequences of Water Infiltrated Soils (part 1 of 2)
Source: PLoS One. 2015 Jan 20;10(1):e0115218. doi: 10.1371/journal.pone.0115218 (PMC4300084; doi:10.1371/journal.pone.0115218)

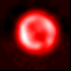

Supplement: S1 File — (ZIP) [file pone.0115218.s001.zip › TomoSolos/PC1/PC1_I_Infiltra_1.bmp]

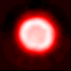

Supplement: S1 File — (ZIP) [file pone.0115218.s001.zip › TomoSolos/PC1/PC1_I_Infiltra_10.bmp]

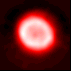

Supplement: S1 File — (ZIP) [file pone.0115218.s001.zip › TomoSolos/PC1/PC1_I_Infiltra_100.bmp]

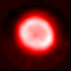

Supplement: S1 File — (ZIP) [file pone.0115218.s001.zip › TomoSolos/PC1/PC1_I_Infiltra_101.bmp]

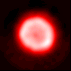

Supplement: S1 File — (ZIP) [file pone.0115218.s001.zip › TomoSolos/PC1/PC1_I_Infiltra_102.bmp]

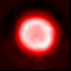

Supplement: S1 File — (ZIP) [file pone.0115218.s001.zip › TomoSolos/PC1/PC1_I_Infiltra_103.bmp]

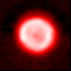

Supplement: S1 File — (ZIP) [file pone.0115218.s001.zip › TomoSolos/PC1/PC1_I_Infiltra_104.bmp]

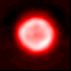

Supplement: S1 File — (ZIP) [file pone.0115218.s001.zip › TomoSolos/PC1/PC1_I_Infiltra_105.bmp]

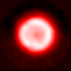

Supplement: S1 File — (ZIP) [file pone.0115218.s001.zip › TomoSolos/PC1/PC1_I_Infiltra_106.bmp]

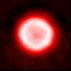

Supplement: S1 File — (ZIP) [file pone.0115218.s001.zip › TomoSolos/PC1/PC1_I_Infiltra_107.bmp]

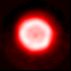

Supplement: S1 File — (ZIP) [file pone.0115218.s001.zip › TomoSolos/PC1/PC1_I_Infiltra_108.bmp]

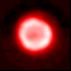

Supplement: S1 File — (ZIP) [file pone.0115218.s001.zip › TomoSolos/PC1/PC1_I_Infiltra_109.bmp]

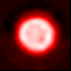

Supplement: S1 File — (ZIP) [file pone.0115218.s001.zip › TomoSolos/PC1/PC1_I_Infiltra_11.bmp]

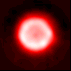

Supplement: S1 File — (ZIP) [file pone.0115218.s001.zip › TomoSolos/PC1/PC1_I_Infiltra_110.bmp]

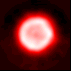

Supplement: S1 File — (ZIP) [file pone.0115218.s001.zip › TomoSolos/PC1/PC1_I_Infiltra_111.bmp]

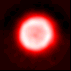

Supplement: S1 File — (ZIP) [file pone.0115218.s001.zip › TomoSolos/PC1/PC1_I_Infiltra_112.bmp]

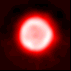

Supplement: S1 File — (ZIP) [file pone.0115218.s001.zip › TomoSolos/PC1/PC1_I_Infiltra_113.bmp]

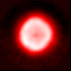

Supplement: S1 File — (ZIP) [file pone.0115218.s001.zip › TomoSolos/PC1/PC1_I_Infiltra_114.bmp]

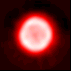

Supplement: S1 File — (ZIP) [file pone.0115218.s001.zip › TomoSolos/PC1/PC1_I_Infiltra_115.bmp]

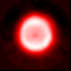

Supplement: S1 File — (ZIP) [file pone.0115218.s001.zip › TomoSolos/PC1/PC1_I_Infiltra_116.bmp]

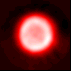

Supplement: S1 File — (ZIP) [file pone.0115218.s001.zip › TomoSolos/PC1/PC1_I_Infiltra_117.bmp]

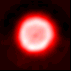

Supplement: S1 File — (ZIP) [file pone.0115218.s001.zip › TomoSolos/PC1/PC1_I_Infiltra_118.bmp]

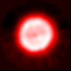

Supplement: S1 File — (ZIP) [file pone.0115218.s001.zip › TomoSolos/PC1/PC1_I_Infiltra_119.bmp]

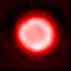

Supplement: S1 File — (ZIP) [file pone.0115218.s001.zip › TomoSolos/PC1/PC1_I_Infiltra_12.bmp]

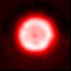

Supplement: S1 File — (ZIP) [file pone.0115218.s001.zip › TomoSolos/PC1/PC1_I_Infiltra_120.bmp]

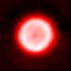

Supplement: S1 File — (ZIP) [file pone.0115218.s001.zip › TomoSolos/PC1/PC1_I_Infiltra_121.bmp]

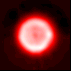

Supplement: S1 File — (ZIP) [file pone.0115218.s001.zip › TomoSolos/PC1/PC1_I_Infiltra_122.bmp]

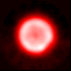

Supplement: S1 File — (ZIP) [file pone.0115218.s001.zip › TomoSolos/PC1/PC1_I_Infiltra_123.bmp]

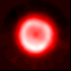

Supplement: S1 File — (ZIP) [file pone.0115218.s001.zip › TomoSolos/PC1/PC1_I_Infiltra_124.bmp]

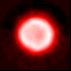

Supplement: S1 File — (ZIP) [file pone.0115218.s001.zip › TomoSolos/PC1/PC1_I_Infiltra_13.bmp]

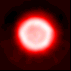

Supplement: S1 File — (ZIP) [file pone.0115218.s001.zip › TomoSolos/PC1/PC1_I_Infiltra_14.bmp]

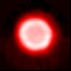

Supplement: S1 File — (ZIP) [file pone.0115218.s001.zip › TomoSolos/PC1/PC1_I_Infiltra_15.bmp]

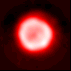

Supplement: S1 File — (ZIP) [file pone.0115218.s001.zip › TomoSolos/PC1/PC1_I_Infiltra_16.bmp]

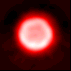

Supplement: S1 File — (ZIP) [file pone.0115218.s001.zip › TomoSolos/PC1/PC1_I_Infiltra_17.bmp]

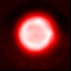

Supplement: S1 File — (ZIP) [file pone.0115218.s001.zip › TomoSolos/PC1/PC1_I_Infiltra_18.bmp]

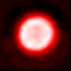

Supplement: S1 File — (ZIP) [file pone.0115218.s001.zip › TomoSolos/PC1/PC1_I_Infiltra_19.bmp]

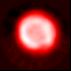

Supplement: S1 File — (ZIP) [file pone.0115218.s001.zip › TomoSolos/PC1/PC1_I_Infiltra_2.bmp]

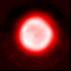

Supplement: S1 File — (ZIP) [file pone.0115218.s001.zip › TomoSolos/PC1/PC1_I_Infiltra_20.bmp]

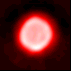

Supplement: S1 File — (ZIP) [file pone.0115218.s001.zip › TomoSolos/PC1/PC1_I_Infiltra_21.bmp]

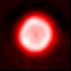

Supplement: S1 File — (ZIP) [file pone.0115218.s001.zip › TomoSolos/PC1/PC1_I_Infiltra_22.bmp]

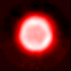

Supplement: S1 File — (ZIP) [file pone.0115218.s001.zip › TomoSolos/PC1/PC1_I_Infiltra_23.bmp]

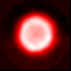

Supplement: S1 File — (ZIP) [file pone.0115218.s001.zip › TomoSolos/PC1/PC1_I_Infiltra_24.bmp]

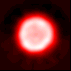

Supplement: S1 File — (ZIP) [file pone.0115218.s001.zip › TomoSolos/PC1/PC1_I_Infiltra_25.bmp]

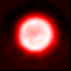

Supplement: S1 File — (ZIP) [file pone.0115218.s001.zip › TomoSolos/PC1/PC1_I_Infiltra_26.bmp]

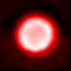

Supplement: S1 File — (ZIP) [file pone.0115218.s001.zip › TomoSolos/PC1/PC1_I_Infiltra_27.bmp]

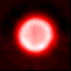

Supplement: S1 File — (ZIP) [file pone.0115218.s001.zip › TomoSolos/PC1/PC1_I_Infiltra_28.bmp]

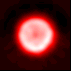

Supplement: S1 File — (ZIP) [file pone.0115218.s001.zip › TomoSolos/PC1/PC1_I_Infiltra_29.bmp]

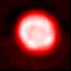

Supplement: S1 File — (ZIP) [file pone.0115218.s001.zip › TomoSolos/PC1/PC1_I_Infiltra_3.bmp]

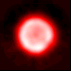

Supplement: S1 File — (ZIP) [file pone.0115218.s001.zip › TomoSolos/PC1/PC1_I_Infiltra_30.bmp]

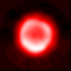

Supplement: S1 File — (ZIP) [file pone.0115218.s001.zip › TomoSolos/PC1/PC1_I_Infiltra_31.bmp]

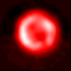

Supplement: S1 File — (ZIP) [file pone.0115218.s001.zip › TomoSolos/PC1/PC1_I_Infiltra_32.bmp]

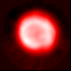

Supplement: S1 File — (ZIP) [file pone.0115218.s001.zip › TomoSolos/PC1/PC1_I_Infiltra_33.bmp]

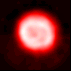

Supplement: S1 File — (ZIP) [file pone.0115218.s001.zip › TomoSolos/PC1/PC1_I_Infiltra_34.bmp]

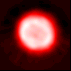

Supplement: S1 File — (ZIP) [file pone.0115218.s001.zip › TomoSolos/PC1/PC1_I_Infiltra_35.bmp]

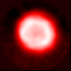

Supplement: S1 File — (ZIP) [file pone.0115218.s001.zip › TomoSolos/PC1/PC1_I_Infiltra_36.bmp]

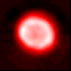

Supplement: S1 File — (ZIP) [file pone.0115218.s001.zip › TomoSolos/PC1/PC1_I_Infiltra_37.bmp]

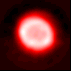

Supplement: S1 File — (ZIP) [file pone.0115218.s001.zip › TomoSolos/PC1/PC1_I_Infiltra_38.bmp]

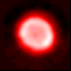

Supplement: S1 File — (ZIP) [file pone.0115218.s001.zip › TomoSolos/PC1/PC1_I_Infiltra_39.bmp]

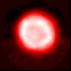

Supplement: S1 File — (ZIP) [file pone.0115218.s001.zip › TomoSolos/PC1/PC1_I_Infiltra_4.bmp]

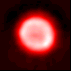

Supplement: S1 File — (ZIP) [file pone.0115218.s001.zip › TomoSolos/PC1/PC1_I_Infiltra_40.bmp]

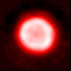

Supplement: S1 File — (ZIP) [file pone.0115218.s001.zip › TomoSolos/PC1/PC1_I_Infiltra_41.bmp]

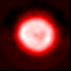

Supplement: S1 File — (ZIP) [file pone.0115218.s001.zip › TomoSolos/PC1/PC1_I_Infiltra_42.bmp]

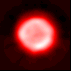

Supplement: S1 File — (ZIP) [file pone.0115218.s001.zip › TomoSolos/PC1/PC1_I_Infiltra_43.bmp]

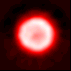

Supplement: S1 File — (ZIP) [file pone.0115218.s001.zip › TomoSolos/PC1/PC1_I_Infiltra_44.bmp]

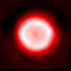

Supplement: S1 File — (ZIP) [file pone.0115218.s001.zip › TomoSolos/PC1/PC1_I_Infiltra_45.bmp]

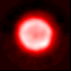

Supplement: S1 File — (ZIP) [file pone.0115218.s001.zip › TomoSolos/PC1/PC1_I_Infiltra_46.bmp]

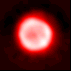

Supplement: S1 File — (ZIP) [file pone.0115218.s001.zip › TomoSolos/PC1/PC1_I_Infiltra_47.bmp]

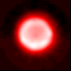

Supplement: S1 File — (ZIP) [file pone.0115218.s001.zip › TomoSolos/PC1/PC1_I_Infiltra_48.bmp]

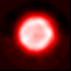

Supplement: S1 File — (ZIP) [file pone.0115218.s001.zip › TomoSolos/PC1/PC1_I_Infiltra_49.bmp]

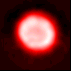

Supplement: S1 File — (ZIP) [file pone.0115218.s001.zip › TomoSolos/PC1/PC1_I_Infiltra_5.bmp]

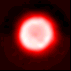

Supplement: S1 File — (ZIP) [file pone.0115218.s001.zip › TomoSolos/PC1/PC1_I_Infiltra_50.bmp]

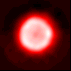

Supplement: S1 File — (ZIP) [file pone.0115218.s001.zip › TomoSolos/PC1/PC1_I_Infiltra_51.bmp]

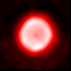

Supplement: S1 File — (ZIP) [file pone.0115218.s001.zip › TomoSolos/PC1/PC1_I_Infiltra_52.bmp]

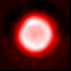

Supplement: S1 File — (ZIP) [file pone.0115218.s001.zip › TomoSolos/PC1/PC1_I_Infiltra_53.bmp]

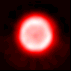

Supplement: S1 File — (ZIP) [file pone.0115218.s001.zip › TomoSolos/PC1/PC1_I_Infiltra_54.bmp]

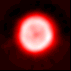

Supplement: S1 File — (ZIP) [file pone.0115218.s001.zip › TomoSolos/PC1/PC1_I_Infiltra_55.bmp]

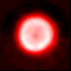

Supplement: S1 File — (ZIP) [file pone.0115218.s001.zip › TomoSolos/PC1/PC1_I_Infiltra_56.bmp]

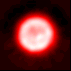

Supplement: S1 File — (ZIP) [file pone.0115218.s001.zip › TomoSolos/PC1/PC1_I_Infiltra_57.bmp]

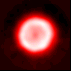

Supplement: S1 File — (ZIP) [file pone.0115218.s001.zip › TomoSolos/PC1/PC1_I_Infiltra_58.bmp]

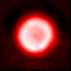

Supplement: S1 File — (ZIP) [file pone.0115218.s001.zip › TomoSolos/PC1/PC1_I_Infiltra_59.bmp]

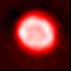

Supplement: S1 File — (ZIP) [file pone.0115218.s001.zip › TomoSolos/PC1/PC1_I_Infiltra_6.bmp]

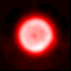

Supplement: S1 File — (ZIP) [file pone.0115218.s001.zip › TomoSolos/PC1/PC1_I_Infiltra_60.bmp]

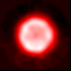

Supplement: S1 File — (ZIP) [file pone.0115218.s001.zip › TomoSolos/PC1/PC1_I_Infiltra_61.bmp]

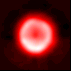

Supplement: S1 File — (ZIP) [file pone.0115218.s001.zip › TomoSolos/PC1/PC1_I_Infiltra_62.bmp]

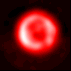

Supplement: S1 File — (ZIP) [file pone.0115218.s001.zip › TomoSolos/PC1/PC1_I_Infiltra_63.bmp]

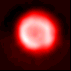

Supplement: S1 File — (ZIP) [file pone.0115218.s001.zip › TomoSolos/PC1/PC1_I_Infiltra_64.bmp]

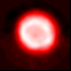

Supplement: S1 File — (ZIP) [file pone.0115218.s001.zip › TomoSolos/PC1/PC1_I_Infiltra_65.bmp]

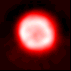

Supplement: S1 File — (ZIP) [file pone.0115218.s001.zip › TomoSolos/PC1/PC1_I_Infiltra_66.bmp]

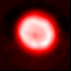

Supplement: S1 File — (ZIP) [file pone.0115218.s001.zip › TomoSolos/PC1/PC1_I_Infiltra_67.bmp]

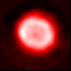

Supplement: S1 File — (ZIP) [file pone.0115218.s001.zip › TomoSolos/PC1/PC1_I_Infiltra_68.bmp]

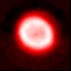

Supplement: S1 File — (ZIP) [file pone.0115218.s001.zip › TomoSolos/PC1/PC1_I_Infiltra_69.bmp]

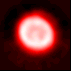

Supplement: S1 File — (ZIP) [file pone.0115218.s001.zip › TomoSolos/PC1/PC1_I_Infiltra_7.bmp]

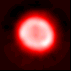

Supplement: S1 File — (ZIP) [file pone.0115218.s001.zip › TomoSolos/PC1/PC1_I_Infiltra_70.bmp]

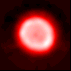

Supplement: S1 File — (ZIP) [file pone.0115218.s001.zip › TomoSolos/PC1/PC1_I_Infiltra_71.bmp]

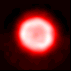

Supplement: S1 File — (ZIP) [file pone.0115218.s001.zip › TomoSolos/PC1/PC1_I_Infiltra_72.bmp]

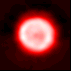

Supplement: S1 File — (ZIP) [file pone.0115218.s001.zip › TomoSolos/PC1/PC1_I_Infiltra_73.bmp]

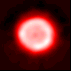

Supplement: S1 File — (ZIP) [file pone.0115218.s001.zip › TomoSolos/PC1/PC1_I_Infiltra_74.bmp]

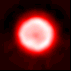

Supplement: S1 File — (ZIP) [file pone.0115218.s001.zip › TomoSolos/PC1/PC1_I_Infiltra_75.bmp]

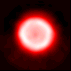

Supplement: S1 File — (ZIP) [file pone.0115218.s001.zip › TomoSolos/PC1/PC1_I_Infiltra_76.bmp]

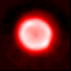

Supplement: S1 File — (ZIP) [file pone.0115218.s001.zip › TomoSolos/PC1/PC1_I_Infiltra_77.bmp]
